# Feasibility, quality and added value of unsupervised at-home spirometry in primary care

**DOI:** 10.1038/s41533-025-00432-y

**Published:** 2025-09-29

**Authors:** T. A. le Rütte, M. Kerkhof, Y. H. Gerritsma, M. M. G. Driessen-Roelfszema, L. van den Bemt, J. W. M. Muris, R. A. Riemersma, H. Sandelowsky, B. Stridh, J. W. H. Kocks

**Affiliations:** 1 General Practitioners Research Institute, Groningen, The Netherlands; 2 Department of Primary and Community Care, Radboud University Medical Center, Nijmegen, The Netherlands; 3 Department of General Practice, Maastricht University, Maastricht, The Netherlands; 4 Department of Family Medicine and Primary Care, Karolinska Institutet, Stockholm, Sweden; 5 Academic Primary Health Care Centre, Region Stockholm, Stockholm, Sweden; 6 KRY Healthcare Center, Stockholm, Sweden; 7 Observational and Pragmatic Research Institute, Singapore, Singapore; 8 Groningen Research Institute Asthma and COPD (GRIAC), University of Groningen, University Medical Center Groningen, Groningen, The Netherlands; 9 Department of Pulmonology, University of Groningen, University Medical Center Groningen, Groningen, The Netherlands

**Keywords:** Asthma, Chronic obstructive pulmonary disease, Diagnosis, Health services

## Abstract

At-home spirometry could provide added value for the diagnosis and monitoring of obstructive pulmonary disease in primary care. However, it is unknown whether implementation in a real-world setting is practicable and produces good quality spirometry. We studied feasibility, quality and added value of at-home spirometry in primary care practices in the Netherlands and Sweden. Adults with an asthma- or COPD-related spirometry indication were provided with equipment to perform unsupervised spirometry at-home. Differences in FEV_1_ and FVC-values from home and general practice were compared, and questionnaires on feasibility were completed by participants and healthcare professionals (HCPs). Of 140 participants, 89.3% completed a home spirometry session, of whom 59.2% produced acceptable spirometry. Overall, HCPs and participants rated home spirometry as feasible and of added value for asthma and COPD monitoring in primary care, though less helpful for diagnostic purposes. A small mean difference in spirometry results was observed, with FEV_1_ and FVC at-home being 0.076 and 0.094 L higher than at the GP office, respectively.

## Introduction

Chronic airway diseases limiting respiratory flow are common, especially asthma and Chronic Obstructive Pulmonary Disease (COPD), with an estimated 300 million and 170 million cases worldwide, respectively^1^. Due to symptomatic similarity, diagnosis remains difficult^2,3^. This can lead to underdiagnosis or misdiagnosis potentially leading to inefficient or incorrect treatment^4^. Chronic airway diseases are diagnosed based on a combination of clinical symptoms and the evidence of lung function abnormalities^5,6^. Spirometry is the key diagnostic tool for these lung function abnormalities. In particular, the presence of obstruction and its reversibility with bronchodilator use are informative for the diagnosis of asthma and COPD and differentiation between them. Therefore, spirometry is essential for the proper diagnosis of airway disease, yet is still underused^7^. Spirometry is also an essential tool for monitoring the progression of airway diseases and is recommended in international guidelines^2,3^. Accompanied by clinical symptom assessment, spirometry provides an objective measure to monitor asthma and COPD progression.

As primary care is usually the first point of contact for patients experiencing respiratory symptoms, the initial diagnosis and subsequent monitoring of asthma and COPD is likely to be assessed by general practitioners (GPs). Providing the GP with accurate information on the patient’s lung function is therefore vital. However, spirometry devices can be bulky, costly and require intensive training to be operated and interpreted. In 2021, most Dutch general practices had access to spirometry, with around 85% performing spirometry in their practice^8^. In 2009, 99% of the Swedish primary care centres (PCC) could utilize spirometry^9^. Performing spirometry in primary care lowers the burden on hospitals, reduces costs and speeds up diagnosis.

A recently introduced restriction on the use of spirometry in primary care resulted from COVID-19. Due to transmission risk, spirometry was temporarily discouraged in general practice in the Netherlands and other parts of the world, making the diagnosis and monitoring of chronic respiratory diseases even more difficult. Although this policy has been reversed in most parts of the world, the imposed spirometry pause and the resulting backlog of unanswered spirometry indications illustrated the need for an alternative to office-based spirometry measurements.

Portable spirometry equipment may offer a solution to the risks associated with COVID-19, because it can be performed at any location. Furthermore, the option to perform spirometry at any time can be more effective to assess trigger-induced asthma than scheduled spirometry at the primary care office or at a diagnostic centre. Asthma is often variable and unpredictable in its manifestation in different patients^10^. Planning a spirometry session to monitor symptom flare-ups, is difficult with often overcrowded primary care schedules. To provide a solution, several mobile spirometry devices have been developed and validated in recent years, including NuvoAir Home^®^^11,12^. Such a device, used together with a smartphone, lacks the need for an interface and computing power and can therefore be small, lightweight, less expensive, and can be used at home compared to traditional spirometers. The international standards for spirometry^13^ have been integrated into the smartphone app that links to the NuvoAir spirometer and are used to judge each session that is performed.

However, while a validated device is an important asset for improving care, proper device usage is equally important^14^. NuvoAir spirometer was validated by comparing it to standard spirometry, while both were operated by the same experienced health care professionals (HCPs)^11^. The way portable spirometry equipment would be used independent of HCPs was not tested in the primary care population. The present study investigates the application of at-home spirometry in adults in the real world. The main challenge with spirometry at home, especially with little or no supervision, is the quality of the measurements. Traditional spirometry requires training for the performing HCPs, which cannot be expected of a patient. Therefore, proper instructions and remote assistance if needed, are of high priority for spirometry at home. If spirometry at home would require more intensive and time-consuming supervision to yield viable results, it may not be feasible for implementation in primary care.

The Spiro@Home study aimed to assess the feasibility, quality, and the added value of at-home spirometry in adult patients for the diagnosis and monitoring of asthma and chronic obstructive pulmonary disease (COPD) in primary care.

## Methods

### Study design

Spiro@Home is a cross-sectional mixed-methods implementation study in general practice that investigates at-home spirometry, using the NuvoAir Air Next® spirometer, coupled with the NuvoAir Home smartphone application. It was approved by the Swedish ethical committee (2022-01563-01) and declared out of scope for the Dutch Medical Research Involving Human Subjects Act by the local ethical committee. Only primary care clinics that perform spirometry in-house and have trained staff, participated in this study. Both spirometry-naïve patients and patients with previous spirometry experience were included. In doing so, we wanted to evaluate at-home spirometry in a broad spectrum of real-world spirometry indications.

In the Spiro@Home study, patients with an asthma- or COPD-related spirometry indication received an at-home spirometry package from their GP’s office. Participants may have been asked to perform both pre- and postbronchodilator spirometry, when indicated for their regular care. The spirometry at home was performed unsupervised. The patients were given written and video instructions that resembled a conventional, supervised spirometry as closely as possible. Spirometry was followed by a self-assessment of symptoms, medication, and lifestyle, which is a regional standard practice^15^. This questionnaire included the asthma control questionnaire (ACQ)^16^, the clinical COPD questionnaire (CCQ)^17^, both of which are reported for the relevant group of participants, and the MRC dyspnoea scale in all subjects^18^, in which a score of 2 or higher was seen as breathlessness being present. This was followed by a second questionnaire about the participant’s experiences with and their acceptance of at-home spirometry. After the at-home spirometry was performed, a standard spirometry was performed at the GP office. This spirometry session was performed under the supervision of trained staff, using their conventional, validated equipment, containing either the Welch-Allyn Spiroperfect or the Carefusion Microlab spirometers. Furthermore, the HCPs completed a questionnaire on their experiences with at-home spirometry, from providing the instructions to interpreting results.

### Study population

Patients were invited to join the Spiro@Home study by their own HCP if there was an asthma- or COPD-related indication to perform spirometry in their medical records. Patients were also invited based on a suspicion of asthma or COPD and their spirometry was considered diagnostic. Participants were included from November 2021 to December 2022. In- and exclusion criteria were limited to ensure an as close to real-world setting as possible. All patients, 16 years or older, with an indication to perform a spirometry session for diagnostic or monitoring purposes of their (possible) asthma or COPD, were eligible. Patients were only excluded at the discretion of the recruiting HCP, based on factors including inability to give informed consent, known inability or contraindications to perform spirometry as listed in the standardization of spirometry 2019 update^13^. HCPs were encouraged not to select or exclude patients based on their age or the HCP’s perception on digital literacy.

### Home spirometry

At home spirometry was performed using the NuvoAir Air Next^®^ handheld spirometer^11,12^, linked to a mobile phone application, installed on a smartphone provided with the equipment. The application provided instructions and feedback on ATS/ERS criteria for the acceptability of the measurements^13^. The participant was asked to perform one spirometry session at home, containing at least three acceptable manoeuvres, with a maximum of eight attempts to achieve this. The software also graded the sessions on an A through F scale based on ATS/ERS criteria, A being the best possible score^13^.

Over the course of the project, it was noticed that test sessions could erroneously be ended and marked as complete by the NuvoAir software already after two acceptable manoeuvres. Sessions in which this happened were scored as grade ‘B’, consistent with the ATS/ERS guidelines. Home spirometry was performed without supervision; therefore, it is unknown how many participants experienced this issue. Participants could close the session themselves, so we could not consider all sessions with two manoeuvres to be affected by the issue. Therefore, for the analysis of the results, both grades ‘A’ and ‘B’ were considered successful.

All at-home spirometry measurements were available via an analytics platform, accessible for both the participant’s GP and the research group. Prints of the results at the GP practice, including the curves, were collected and analysed.

Forced expiratory volume in 1 s (FEV_1_) and forced vital capacity (FVC) were recorded for each manoeuvre together with the highest value achieved.

The best manoeuvre within a session was selected by the software that was used to perform the spirometry. Errors within this best manoeuvre of the at-home spirometry session and office spirometry were assessed by two independent experts who used the following ATS/ERS criteria:

- A slow start, defined as a back-extrapolated volume on the volume-time curve of ≥5% of FVC or 0.100 L, whichever is greater
- Did not reach one of these two end of forced expiration indicators
  1. Expiratory plateau (≤0.025 L in the last 1 s of expiration)
  2. Expiratory time ≥15 s

- No interruption / no evidence of cough or glottis closure in the first second of expiration
- No interruption / no evidence of glottis closure after the first second of expiration

A third expert was consulted in cases of disagreement. To ensure that the experts could not compare the sessions within participants, each session was assigned an identifier separate from participant ID.

### Analysis

Analyses were performed in Rstudio, using R version 4.2.1^19^. Characteristics of the participant population were described as mean values with standard deviations when normally distributed or median values with interquartile range for continuous variables, and numbers and percentages for categorical variables. Characteristics of those who completed at-home spirometry were compared with characteristics of participants who were unable to do so by using chi-squared tests for proportions and Mann-Whitney U tests for continuous variables.

When reversibility testing was indicated, only the pre-bronchodilator session was used for analyses to eliminate the effect of differences in bronchodilator administrations between sessions at-home and at the GP office.

To test the degree of agreement of home spirometry, both FEV_1_ and FVC were compared between home and control sessions. Bland Altman analysis was performed to calculate the bias, including 95% confidence intervals and the limits of agreement, using the ‘blandr’ package^20^. Pearson’s r was calculated for the correlation and a 2-variable linear model was used to estimate the slope and 95% confidence intervals. Linear hypothesis testing was performed to test if the slopes differed from the identity line, using the ‘car’ package^21^. The evaluations from participants and HCPs were plotted using the ‘likert’ package^22^.

## Results

### Characteristics

A total of 140 participants signed the informed consent form, of whom 125 (89.3%) produced a spirometry session. The remaining 15 participants made attempts but did not succeed in recording a spirometry session. The participants were recruited at 32 sites, of which 19 were in the Netherlands. The different sites included between 2 and 11 participants. In general, demographic characteristics were not relevantly different between participants with and without spirometry results (Table S1).

Clinical characteristics obtained by questionnaire were available from 116 participants of whom 106 provided spirometry results (Table 1). Most participants reported a history of asthma, starting before 20 years of age. Around one third of participants reported no history of COPD or asthma. Symptoms of asthma or COPD were on average mild among participants as measured with the ACQ and CCQ (Table 1). Breathlessness (MRC ≥ 2) was reported by 15.4% of participants and 17.5% of the participants experienced one or more exacerbations in the last year.

**Table 1:** Characteristics of participants who have performed home spirometry, obtained by self-reported questionnaire.

Characteristic	Participants
N	125
Sex=Male, n (%)	46 (36.8)
Age, years (median [IQR])	45.0 [32.0, 63.0]
Height, cm (mean (SD))	173.1 (9.4)
BMI, kg/m^2^ (mean (SD))	26.7 (5.3)
Smoking status, available n (%)	106 (84.8)
Current smoker	15 (12.0)
Ex-smoker	40 (32.0)
Never smoker	51 (40.8)
Age of onset of respiratory symptoms, available n (%)	92 (73.6)
Continuous, median [IQR]	19.0 [7.0, 46.2]
Categorical, n (%)	
<20	46 (36.8)
20–39	17 (13.6)
40–59	19 (15.2)
≥60	10 (8.0)
Previously known diagnosis, available n (%)	98 (78.4)
Asthma (%)	50 (40.0)
COPD (%)	9 (7.2)
Asthma & COPD (%)	7 (5.6)
No Asthma or COPD (%)	32 (25.6)
Breathlessness, available n (%)	91 (72.8)
Breathlessness (mMRC≥2) (%)	14 (11.2)
No breathlessness (mMRC<2) (%)	77 (61.6)
CCQ^a^ in patients with COPD, available n (%)	15 (93.8)
Mean (SD)	1.36 (0.8)
ACQ^b^ in patients with asthma, available n (%)	50 (87.7)
Mean (SD)	0.98 (0.7)
Exacerbations last year^c^, available n (%)	92 (73.6)
0 (%)	75 (60.0)
1 (%)	9 (7.2)
≥2 (%)	8 (6.4)

IQR interquartile Range, *SD* standard deviation, *BMI* body mass index.

^a^Clinical COPD questionnaire.

^b^Asthma control questionnaire.

^c^Self-reported question: In the last 12 months, how often did you have an antibiotic or prednisolone course due to increased respiratory symptoms, such as cough and/or shortness of breath?.

### Grading

Successful spirometry, defined as ≥2 acceptable measurements (repeatable within 150 ml) of FEV_1_ and FVC, was obtained from 74 (59.2%) of the 125 participants who delivered home spirometry results (Table 2).

**Table 2:** Distribution of NuvoAir session grading among participants who delivered home spirometry results (*N* = 125).

Grade	A	B	C	D	E	F
N (%)	38 (30.4)	36 (28.8)	9 (7.2)	1 (0.8)	24 (19.2)	17 (13.6)

### Quality comparison

A total of 116 participants performed a spirometry session at home and at the GP office and could therefore be compared on quality. In 53% of these sessions, the third assessor was needed to reassess at least one of the errors, because of disagreement between the first two independent experts. Participants were free of errors more frequently in the home sessions than in the sessions at the GP office (Table 3). In both settings, it was more common for a session to contain one error than multiple errors.

**Table 3:** Distribution of the number of errors made at spirometry sessions at home and at the GP office.

N (%)	0	1	2	3	4
At home	92 (79.3)	14 (12.1)	2 (1.7)	3 (2.6)	5 (4.3)
GP office	81 (69.8)	25 (21.6)	4 (3.4)	6 (5.2)	0 (0)

**Fig. 1:**
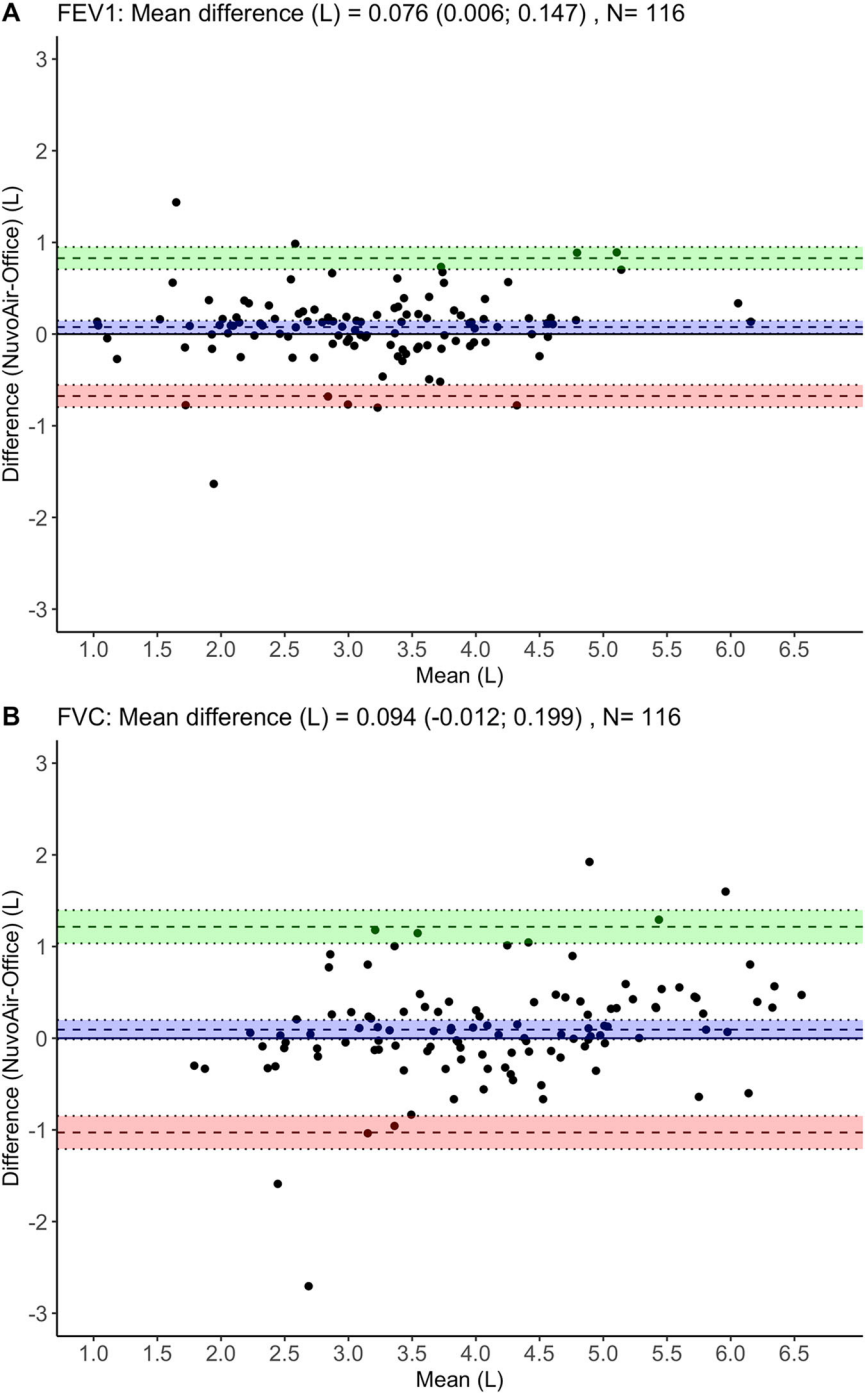
Bland-Altman plots of the agreement between test settings. **A** and FVC **B** values from the NuvoAir home session and measured at the healthcare centre. The x-axis shows the mean value of the two settings and the y-axis shows the difference between the two, with a difference above 0 indicating higher results produced in the NuvoAir spirometry. The dashed lines indicate the mean difference and the upper and lower limits of the 95% confidence intervals (CI).

The mean difference between the best FEV_1_ value measured at home and the best FEV_1_ value from practice spirometry was 0.076 L (95% confidence interval (CI): 0.006; 0.147) (Fig. 1A). The 95% limits of agreement were −0.676 and 0.828. There was a strong correlation between both FEV_1_ values (r = 0.932, 95% CI: 0.904; 0.953), and no evidence of the slope significantly deviating from the identity line, with a slope of 0.972 (95% CI: 0.903; 1.042, *p* = 0.44) (Fig. S1).

The mean difference between the best FVC value measured at home and the best FVC value from practice spirometry was 0.094 L (95% CI: −0.012; 0.199) (Fig. 1B). The 95% limits of agreement were −1.028 and 1.216. The correlation between both FVC values was good (r = 0.884, 95% CI: 0.836; 0.918), and no evidence of the slope significantly deviating from the identity line, with a slope of 1.023 (95% CI: 0.923; 1.124, *p* = 0.65) (Fig. S1).

### Feasibility and added value

#### Healthcare professional questionnaire

**Fig. 2:**
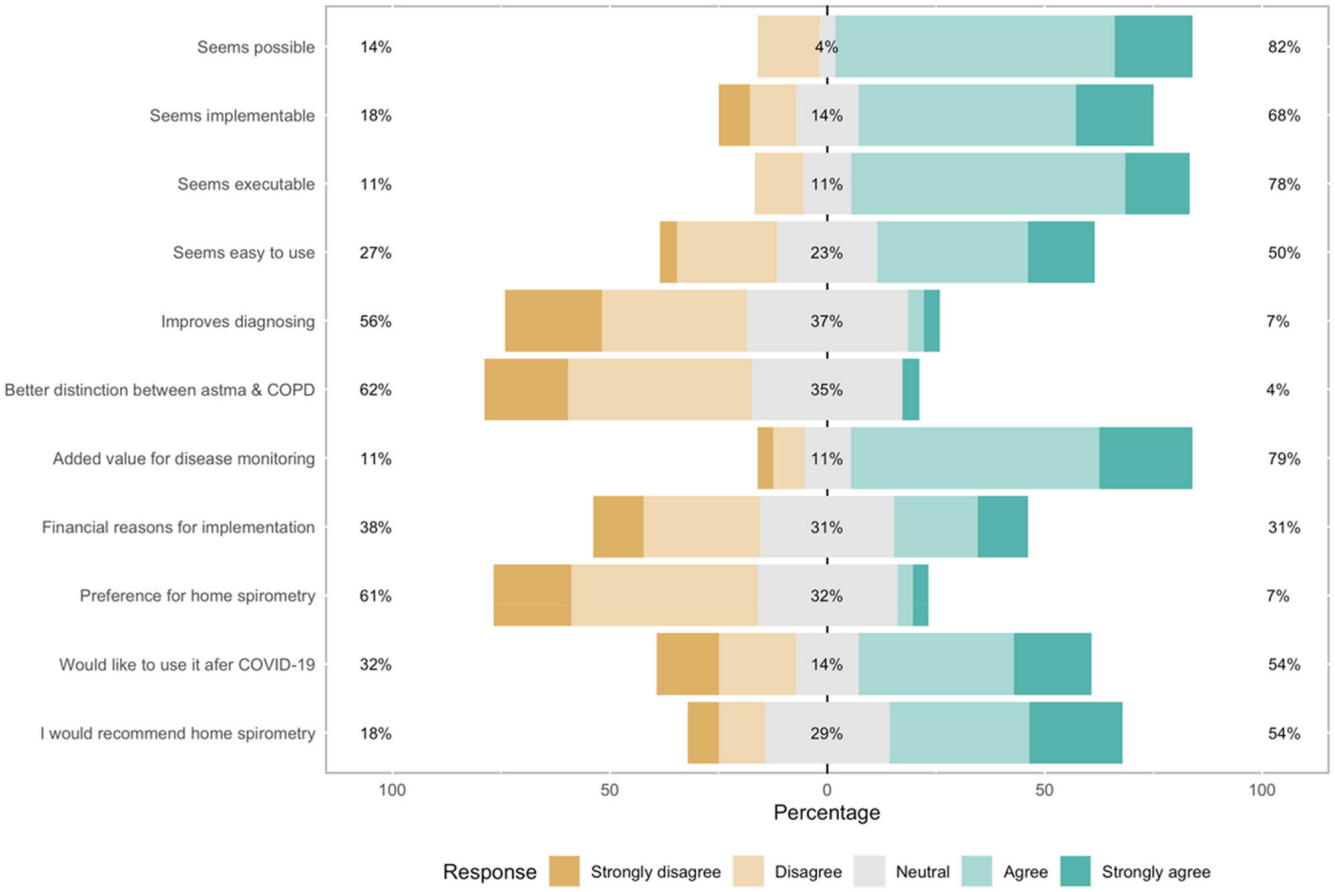
Feasibility of performing spirometry at home using NuvoAir rated on a Likert-scale by general practice spirometry professionals (*N* = 28).

At-home spirometry was evaluated by 28 HCPs (24 practice nurses, 4 GPs) through a questionnaire. The majority found home spirometry implementable (68%), possible (82%), and executable (78%) (Fig. 2). The use of home spirometry was not seen as an improvement to the diagnostic process (7%). However, 79% of professionals saw added value of at-home spirometry for disease monitoring. After using it in their practice, half of the HCPs (54%) would recommend home spirometry and would like to keep using it after the COVID-19 pandemic.

#### Participant questionnaire

**Fig. 3:**
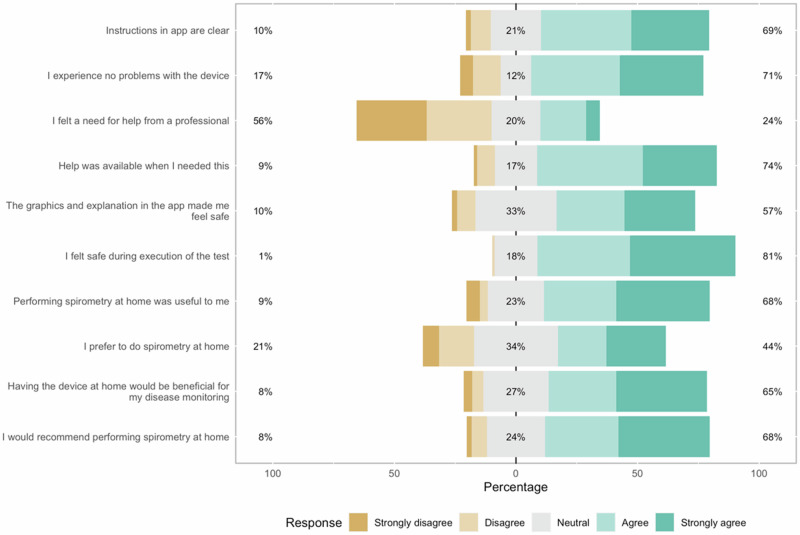
Responses of participants to the questions on their experiences with home spirometry (*N* = 101).

The participant questionnaires were filled in by 101 participants (Fig. 3). The majority of these participants found it useful (68%), found it beneficial for their disease monitoring (65%) and would recommend performing spirometry at home to others (68%). Most participants indicated that they did not feel the need for assistance from a medical professional, and 71% experienced no problems. Home spirometry was preferred over spirometry at a GP office by 44% of participants.

## Discussion

The current study evaluated at-home spirometry for quality, feasibility and added value for the diagnosis and monitoring of asthma and COPD in primary care. The main finding of this study was that 59.2% of the participants who recorded an at-home spirometry session produced good quality results. Also, implementation of home spirometry was evaluated to be feasible in a substantial part of the population of patients with obstructive lung disease, but less so for diagnostic use.

Comparison of the absolute spirometry values obtained from NuvoAir with those produced at the GP office showed evidence of FEV_1_ values produced at home being slightly higher than those measured at the GP office. No significant mean difference was found for FVC. However, a large spread in within-participant differences was found.

Participants who responded to the evaluation questionnaire were generally happy with performing spirometry at home and saw added value. Healthcare professionals were also generally positive. Whilst they do not think spirometry at home to be better suited for diagnosis or for distinguishing asthma for COPD, they did see added value for disease monitoring.

Similar performance based on error judgement, good correlation coefficients and a small bias towards higher absolute values indicate that the quality of the measurements performed at home is similar to the spirometry at the GP office. Few studies comparing at-home spirometry to office spirometry have been published. In one similar study in patients with cystic fibrosis, participants performed comparably at-home and at the GP^22,23^.

There is no obvious explanation for the NuvoAir application having produced higher absolute values of FEV_1_as compared with the healthcare centre results. This finding is inconsistent with earlier research validating the NuvoAir Air Next device^11,24,25^. A recent real-world study found no differences in absolute values between unsupervised NuvoAir spirometers used by people with cystic fibrosis and in-clinic spirometry with favourable participant experience^26^. Therefore, it is unlikely to result from the NuvoAir device and the software behind it. We can only speculate about reasons for healthcare centre values being slightly lower. One of the reasons might be that people feel more comfortable to take the time to fully exhale if no one is present.

The presence of a rather large overall spread in the differences illustrates the difficulty of producing consistent spirometry results across sessions. The sessions were scheduled as close to each other as possible, usually several days apart. Although both sessions were planned at approximately the same time of day for each participant, the fact that there were several days between sessions could partially explain a large spread.

Lower quality of the manoeuvres would generally result in lower outcomes, because of the nature of the test. However, sub-optimal peak expiratory flow (PEF) could lead to both over- and underestimation of FEV_1_. Therefore, spirometry software could select manoeuvres with sub-optimal PEF with higher FEV_1_ as a best effort, while not being the maximal effort that the guidelines require. While this was not exclusive to the NuvoAir software, personal coaching could result in better overall technique, as a sub-maximal effort is often poorly identified by software^27^.

The small difference in lung function parameters and smaller proportion of errors at home compared to sessions at the GP office indicate that spirometry performed at home does not lead to a lower quality of results. At-home spirometry would allow the patient the possibility to perform a spirometry session on multiple consecutive days, without increasing the burden on the HCPs. This could enable a way to increase the accuracy of the results to represent the actual situation of the patients’ lung function. An earlier study on a multiple-day follow-up with home spirometry in patients with asthma showed that a majority of patients were able to produce consistently acceptable results^28^. However, the participants in this earlier study received more intensive training and no clinical values were reported. Therefore, an interesting suggestion for future research would be to study the development of clinical values over time, with little initial training. This could potentially show a learning effect and give an indication of an ideal timeframe for a multiple-day follow-up.

Home spirometry was not consistently preferred over office spirometry, although it was received positively and seemed implementable.

In general, the results suggest that most added value for home spirometry in primary care can be found in monitoring of disease progression rather than part of diagnostic procedures. Patients who are monitored have become familiar with the effort and technique that are needed to perform a successful test and are more likely to apply that when testing without supervision.

The answers to the questionnaires suggest that HCPs have significant doubts about using home spirometry for diagnosis, with patients that are inexperienced with spirometry. The HCPs were instructed to recruit patients with varying experience, which they have done, as indicated by one third of the participants reporting neither asthma nor COPD as a diagnosis prior to the study. The participants who reported no previous diagnosis had a diagnostic indication for spirometry and therefore had little experience. The relatively negative feedback by the HCPs about the diagnostic potential of home spirometry show that supervision may still be needed for this group, and that home spirometry will not be able to completely replace conventional spirometry for asthma or COPD care.

Even without considering the diagnostic purpose of spirometry, the added value of a more flexible solution for monitoring asthma or COPD should not be overlooked. While the variability of asthma symptoms is well known, deterioration of FEV_1_ can predict exacerbations up to two weeks in advance, even in patients with COPD^29^. When combined with digital self-management solutions^30^ or alerts to the physician, early detection of symptom flare-ups could help patients to better control their pulmonary disease.

Within this study, some practical issues with home spirometry came to light, as 15 participants were not able to successfully deliver a spirometry session. Even though some participants indicated experiencing problems with the device, the available options for support, such as email and phone, were not used by the participants. Patients may have been more likely to contact their own physician or attempt to resolve problems without help than contact the available helpdesk. However, opportunities for intensive technical support may not be equally available in the real-world implementation of at-home spirometry. Therefore, the lack of technical support use may have increased start-up problems for participants and healthcare centres, but it also simulated a real-world situation more accurately.

Participants received little training before the test, relying mostly on written instructions and short videos. This differs from earlier research on home spirometry^23,31^, but will more likely resemble the training patients with asthma or COPD would receive in daily care. Although asthma and COPD patients at best only perform spirometry annually, giving little opportunity or cause for extensive training, technical problems might become less likely with consecutive sessions. While more intensive training may be feasible for patients with CF, long term use by a healthcare organization would lead to repetition in patients with asthma or COPD, enabling easier start-up and testing.

Several sites experienced technical problems with the NuvoAir app and its provider portal. The most notable problem was poor connectivity. In the current study, the sites received a smartphone with the NuvoAir Home app to ensure uniformity and to reduce the burden of having to download an app on participants’ personal phones. However, having to work with an unfamiliar device and connect to the home Wi-Fi network may have increased the technical difficulty and burden more than expected. Patients can download the app on their own device, this flexibility may alleviate some of the technical burden during real-world implementation. A mobile internet connection would also help reduce connectivity problems when giving a phone with the spirometer.

The software occasionally ending the session after two accepted manoeuvres limited the accuracy of the software’s grading. According to ATS/ERS spirometry guidelines^13^, three manoeuvres are needed to assess the acceptability of a spirometry session, so repeatability can be used as a criterion. The sessions in which no third acceptable manoeuvre could be attempted, were graded ‘B’ by default, therefore the distinction between sessions graded ‘A’ and ‘B’ cannot be made objectively.

For control spirometry sessions, full ATS/ERS grading of the control spirometry could not be done because the data of individual spirometry efforts were not always available. Part of the sites provided the best efforts only. Therefore, the repeatability could not be assessed properly.

Although all patients with a spirometry indication related to asthma or COPD were eligible for inclusion, HCPs were instructed to include patients that they would normally ask to perform spirometry at-home. Therefore, patients with limited computer skills may have been selectively excluded from participation, likely resulting in the exclusion of part of the elderly patient population. Probably partly due to this indirect selection by age, COPD patients were underrepresented. It is possible that home spirometry is less suitable for this relatively older population, which is generally more affected by digitalisation in healthcare. The current validation results do not apply to all patients undergoing spirometry in general practice, however, the authors believe that it fits an implementation study well, as it simulated how HCPs would select patients for home-based spirometry in a real-world setting.

## Conclusion

Implementation of home spirometry is feasible for measuring diagnosed or suspected obstructive lung disease in primary care. However, not all participants were able to produce valid results. We found no evidence of lower quality results from home spirometry compared with spirometry produced under the guidance of a healthcare professional. Questionnaires on feasibility and added value submitted by HCPs and participants showed that both parties recognised added value of home spirometry in primary care, especially when applied for disease monitoring.

## Supplementary information

Supplementary Information

## Data Availability

The datasets used and analysed during the current study are available from the corresponding author on reasonable request.
