# Supplementary material for: Feasibility, quality and added value of unsupervised at-home spirometry in primary care
Source: NPJ Prim Care Respir Med. 2025 Sep 29;35:42. doi: 10.1038/s41533-025-00432-y (PMC12480718; doi:10.1038/s41533-025-00432-y)
Supplement: Supplementary file 1 — Supplementary Information [file 41533_2025_432_MOESM1_ESM.docx]

Supplements

Table S1: Self-reported questionnaire on clinical characteristics

| Characteristic | Included to participate | Performed at-home spirometry |
| --- | --- | --- |
| N | 140 | 125 |
| Sex = Male, n (%) | 56 (40.3) | 46 (36.8) |
| Age, years (median [IQR]) | 46.0 [33.0, 63.0] | 45.0 [32.0, 63.0] |
| Height, cm (mean (SD)) | 173.1 (9.2) | 173.1 (9.4) |
| BMI, kg/m^2^ (mean (SD)) | 26.5 (5.1) | 26.7 (5.3) |
| Smoking status, available n (%) | 117 | 106 |
| Current smoker | 17 (14.5) | 15 (14.2) |
| Ex-smoker | 45 (38.5) | 40 (37.7) |
| Never smoker | 55 (47.0) | 51 (48.1) |
| Age of onset of respiratory symptoms, available n (%) | 102 (72.9) | 92 (73.6) |
| Continuous, median [IQR] | 20.0 [7.0, 50.0] | 19.0 [7.0, 46.2] |
| Categorical, n (%) |  |  |
| <20 | 49 (48.0) | 46 (50.0) |
| 20-39 | 18 (17.6) | 17 (18.5) |
| 40-59 | 22 (21.6) | 19 (20.7) |
| ≥60 | 13 (12.7) | 10 (10.9) |
| Previously known diagnosis, available n (%) | 109 (77.9) | 98 (78.4) |
| Asthma (%) | 55 (50.5) | 50 (51.0) |
| COPD (%) | 13 (11.9) | 9 (9.2) |
| Asthma & COPD (%) | 7 (6.4) | 7 (7.1) |
| No Asthma or COPD (%) | 34 (31.2) | 32 (32.7) |
| Breathlessness, available n (%) | 100 (71.4) | 91 (72.8) |
| Breathlessness (mMRC≥2) (%) | 17 (17.0) | 14 (15.4) |
| No breathlessness (mMRC<2) (%) | 83 (54.4) | 77 (61.6) |
| CCQ^a^ in patients with COPD, available n (%) | 19 (95.0) | 15 (93.8) |
| Mean (SD) | 1.5 (0.8) | 1.4 (0.8) |
| ACQ^b^ in patients with asthma, available n (%) | 51 (82.3) | 50 (87.7) |
| Mean (SD) | 1.0 (0.7) | 1.0 (0.7) |
| Exacerbations previous year^c^, available n (%) | 102 (72.9) | 92 (73.6) |
| 0 (%) | 84 (82.4) | 75 (81.5) |
| 1 (%) | 10 (9.8) | 9 (9.8) |
| ≥2 (%) | 8 (7.8) | 8 (8.7) |

IQR=Interquartile Range; SD= Standard Deviation; BMI=Body Mass Index

^a^ Clinical COPD questionnaire

^b^ Asthma control questionnaire

^c^ Self-reported question: In the last 12 months, how often did you have an antibiotic or prednisolone course due to increased respiratory symptoms, such as cough and/or shortness of breath?

Table S2. Distribution of errors made in sessions, as assessed by independent professionals.

|  | **≥1 error in both sessions** | **≥1 error in office session only** | **≥1 error in home session only** | **No errors either session** |
| --- | --- | --- | --- | --- |
| **Back-extrapolation (N=116)** | 2 (1.7%) | 8 (6.8%) | 8 (6.8%) | 98 (84.4%) |
| **End of forced expiration indicators (N=115)** | 4 (3.4%) | 13 (11.3%) | 14 (12.2%) | 84 (73.0%) |
| **No interruption in the first second (N=116)** | 2 (1.7%) | 6 (5.2%) | 7 (6.0%) | 101 (87.1%) |
| **No interruption after the first second (N=116)** | 3 (2.6%) | 15 (12.9%) | 7 (6.0%) | 91 (78.4%) |
| **Any of the above errors**  **(N=116)** | 10 (8.8%) | 25 (22.1%) | 13 (11.5%) | 65 (57.5%) |


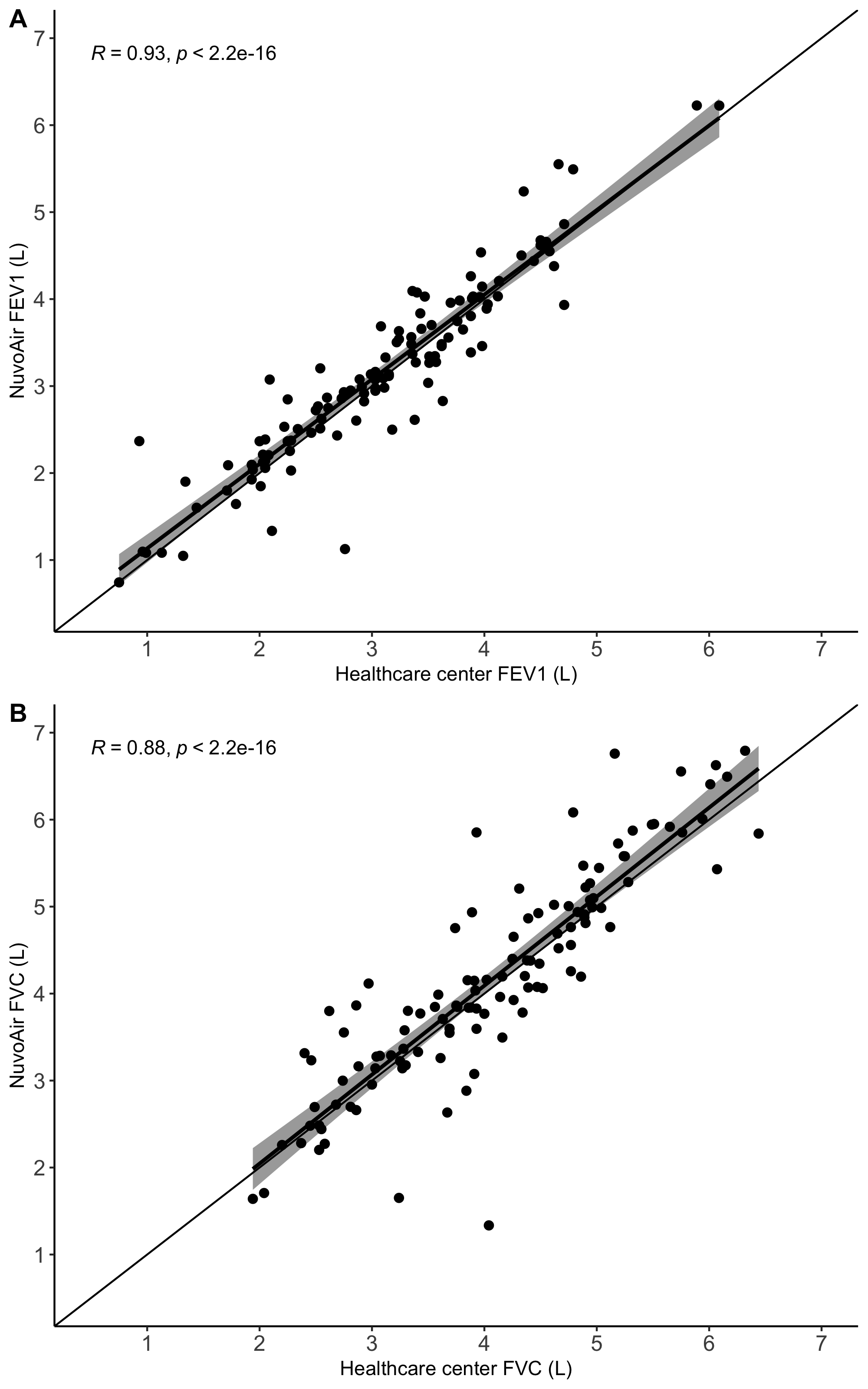


Figure S1. Correlation plots of the agreement between the best FEV_1_ (A) and FVC (B) values from the NuvoAir home session and obtained from GP office spirometry.
